# Factors contributing to the delayed diagnosis of endometriosis—a systematic review and meta-analysis

**DOI:** 10.3389/fmed.2025.1576490

**Published:** 2025-07-22

**Authors:** Wenwei Li, Huiyan Feng, Qingjian Ye

**Affiliations:** Department of Gynaecology, The Third Affiliated Hospital of Sun Yat-sen University, Guangzhou, China

**Keywords:** endometriosis, diagnostic delay, meta-analysis, patient-related factors, provider-related factors, systematic review, misdiagnosis, healthcare disparities

## Abstract

**Background:**

Endometriosis is a prevalent gynecological disorder that is estimated to affect approximately 10% of women of childbearing age globally. However, the condition remains significantly under-or misrecognized, and the mean time to diagnosis is several years. These delays result in increased symptom severity, diminished quality of life, and adverse long-term reproductive outcomes. Therefore, the aim of this systematic review and meta-analysis was to estimate the sources of diagnostic delay and their effects.

**Methods:**

The articles were retrieved from PubMed, Cochrane, and Google Scholar databases after performing a rigorous literature search. The initial search yielded 2,348 records, and 10 articles were included in the final analysis. Causes of diagnostic delay were classified under patient, physician, and systems attributes. Random-effects analysis was used to estimate the overall SMDs with 95% confidence intervals (CIs). For the primary *post hoc* analysis, subgroup analyses and evaluation of heterogeneity (*I*^2^ statistic) were conducted. Publication bias was checked using Egger’s test and funnel plots.

**Results:**

Patient-related factors demonstrated a significant pooled effect size (SMD: 1.94, 95% CI: 1.62–2.27, *p* < 0.001), with delays in seeking medical attention (SMD: 2.14, 95% CI: 1.36–2.92) contributing most prominently. Provider-related factors, including misdiagnosis and reliance on non-specific diagnostics, also had a substantial pooled effect size (SMD: 2.00, 95% CI: 1.72–2.28, *p* < 0.001), with low heterogeneity (*I*^2^ = 3%). System-related factors, such as referral pathways and geographic disparities, were not analyzed separately due to insufficient data for the subgroup. Funnel plot analysis showed no significant publication bias (Egger’s test *p* = 0.57).

**Conclusion:**

The results indicate that diagnostic delays in endometriosis are a function of multiple factors, with key contributions from patient and provider-related barriers. To close these gaps, targeted interventions such as public education to combat symptom normalization and stigmatization, more comprehensive provider training, and streamlined diagnostic pathways are needed. Future research can evaluate system-level changes and non-invasive diagnostic tools to reduce systemic delays further.

## Introduction

Endometriosis is a non-cancerous disease with inflammatory components and affects 6–10% of women of childbearing age (1). Endometriosis is a condition where endometrial tissue develops outside the uterus and can cause dysmenorrhea, chronic pelvic pain, dyspareunia, and infertility (2, 3). However, endometriosis remains a disease that is not well understood by many and is often confused with other illnesses affecting millions of women globally and not afforded the correct treatment (4). Moreover, the consequences of such a delay extend to not only physical but also psychological aspects, affecting quality of life and even socioeconomic aspects (5).

The diagnostic challenge associated with endometriosis stems from its diverse and non-specific manifestations and the fact that it may mimic other conditions such as irritable bowel syndrome (IBS) and pelvic inflammatory disease (PID) (6, 7). This diagnostic difficulty is, however, compounded by social and cultural factors such as the acceptance of dysmenorrhea and the social taboos that accompany discussion on menstruation (8). Primary care physicians, including general practitioners (GPs), often overlook endometriosis-related symptoms or misdiagnose them as other benign or unrelated illnesses (9). Diagnostic procedures, like laparoscopy, which are also accurate, contribute to the delays because they require specialists and are often performed when the cases are already advanced (10).

Some of the challenges highlighted in recent research include the complex healthcare system, lack of a proper referral system, and unequal access to healthcare (11). Geographic factors are also highly relevant, whereby diagnostic delays are found to be more significant in areas that lack specialized and/or diagnostic facilities (12, 13). Although there has been some improvement with newer imaging technology and growing awareness among clinicians, deficiencies still exist that require addressing and understanding the causes of these delays (14).

The delay in disease diagnosis not only worsens the disease but creates a domino effect of negative consequences, including increased healthcare costs, lost productivity, and higher psychological impact (15, 16). Furthermore, delayed diagnosis has serious consequences on reproductive health because if endometriosis is left untreated, it can lead to infertility and pregnancy complications (17). These delays can be considered preventable to improve patient satisfaction, reduce the total cost of healthcare, and ensure equal access to timely services (18).

As diagnostic delays are widespread and affect a significant number of individuals, the present systematic review and meta-analysis aim to provide a comprehensive review of the literature regarding the causes of endometriosis diagnostic delays (19, 20). By assessing the patient, provider, and system-level factors that may pose challenges to the diagnostic process, this study aims to identify areas for attention that can enhance the diagnostic process. The purpose of this review is to determine and quantify the extent of diagnostic deferral, to examine the causes of such delays, and to determine the impact on patients’ outcomes, thereby guiding future practice for clinicians, officials, and scholars.

## Methodology

This systematic review and meta-analysis adheres to the Preferred Reporting Items for Systematic Reviews and Meta-Analyses (PRISMA) guidelines. The review process involved conducting a search according to a predetermined method, applying inclusion and exclusion criteria, screening the studies, extracting data, assessing quality, and analyzing the data using statistical software.

### Search strategy

A comprehensive search was conducted across three major databases: PubMed, Cochrane Library, and Google Scholar for publications up to December 2023. Medical Subject Headings (MeSH) terms were used, as well as other specific topics related to endometriosis, diagnostic delay, barriers to diagnosis, and factors influencing the diagnosis. The Boolean terms of conjunction and disjunction, as well as truncation, were employed to enhance the search approach.

The duality of records was eliminated with the help of reference management software. Moreover, the bibliographic databases of the included studies and relevant reviews were hand searched for other studies.

### Inclusion and exclusion criteria

#### Inclusion criteria

1. Studies reporting diagnostic delays in endometriosis.
2. Original research articles, including observational (cross-sectional, case–control, and cohort) and qualitative studies.
3. Studies with a sample size of ≥20 participants.
4. Studies reporting outcomes related to patient, provider, or system-related factors contributing to delays.
5. Articles published in English.

#### Exclusion criteria

1. Studies focusing exclusively on treatment outcomes without discussing diagnostic delays.
2. Case reports, reviews, conference abstracts, and opinion articles.
3. Studies without adequate data on diagnostic timeframes or factors contributing to delays.
4. Non-English language articles.

Although the initial search retrieved 2,348 records, only 10 met our *a priori* PICOS-driven criteria. The stringent exclusion of studies lacking quantitative delay metrics, combined with our decision to restrict analyses to articles reporting ≥20 participants, inevitably narrowed the final pool but ensured methodological comparability and reduced heterogeneity.

### Study screening

The initial search yielded 2,348 records. Titles and abstracts were reviewed and rated for relevance by two authors. Articles that were not relevant to the criteria for inclusion were not considered. The identified full-text articles were then screened and assessed for relevance to the study. Any disagreements between the reviewers were resolved through consensus or with the help of a third reviewer.

### Data extraction

Data from the included studies were extracted using a standardized form. Extracted data included the following:

- Study characteristics: authors, year of publication, country, study design, and sample size.
- Participant demographics: mean age at symptom onset and diagnosis, geographic region.
- Diagnostic delays: total timeframe from symptom onset to diagnosis, delays at different stages (e.g., patient, provider, and system-related delays).
- Factors contributing to delays: patient, provider, and system-related barriers.
- Reported outcomes: quality of life, symptom progression, and reproductive health impacts.

### Quality assessment

The quality of the studies included was assessed using the Newcastle–Ottawa Scale (NOS) which is typically employed for observational studies. The NOS evaluates studies based on three domains: participation criteria, sample similarity, and evaluation of results. For each study, the quality of the methodological approach was measured on a scale of 1–9, with higher values meaning higher quality. In qualitative research, quality was determined by the measures that were already in place, namely credibility, transferability, and dependability. The results of the quality assessment are presented in Table 1.

**Table 1:** Quality assessment of the included studies using NOS.

Study ID	Selection (4 points)	Comparability (2 points)	Outcome (3 points)	Total NOS score
Hadfield et al. (29)	★★★	★	★★	6
van der Zanden et al. (27)	★★★★	★★	★★★	9
Ballard et al. (23)	★★★	★	★★	6
Staal et al. (24)	★★★	★★	★★★	8
Ghai et al. (28)	★★★★	★★	★★★	9
van der Zanden et al. (26)	★★★★	★★	★★	8
van der Zanden et al. (33)	★★★★	★	★★	7
Hudelist et al. (22)	★★★★	★★	★★★	9
Seear (21)	★★★	★	★★	6
Soliman et al. (25)	★★★★	★★	★★★	9

### Data synthesis

The extracted data were described both qualitatively and numerically. For meta-analysis, statistical software R (version 4.2.3)^1^ was used. The random effects model was used in the analysis to address the issue of heterogeneity across the studies. Diagnostic delay was expressed as pooled means and 95% CIs. Additional analyses were conducted by geographic region, study design, and diagnostic technique.

Interstudy heterogeneity was evaluated using the *I*^2^ statistic, where an *I*^2^ > 50% was considered substantial. Publication bias was assessed graphically using funnel plots and statistically using the Egger test. The study results were further evaluated using sensitivity analyses to assess the impact of excluding high-risk-of-bias studies or small sample sizes.

## Results

**Figure 1:**
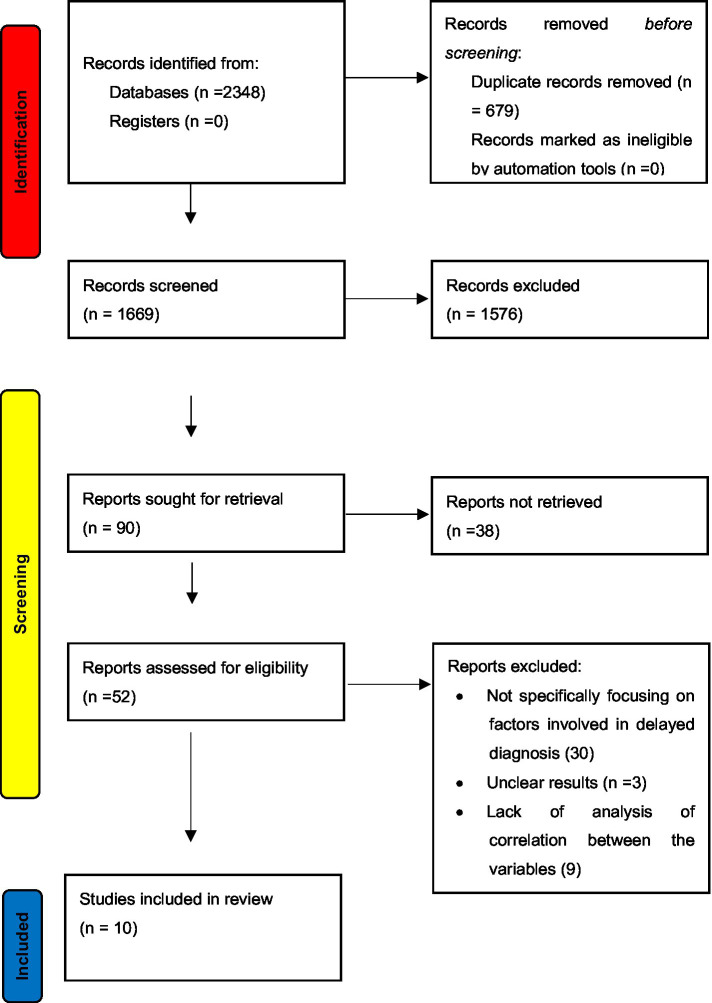
PRISMA flow diagram of included studies.

After performing the exhaustive database search, 2,348 records were retrieved. After removing duplicates and applying the inclusion and exclusion criteria, 10 articles were considered for analysis in this systematic review and meta-analysis. The PRISMA flow diagram is illustrated in Figure 1, which describes the inclusion and exclusion criteria of the articles.

The quality of the included studies was evaluated using the NOS, a scale designed to assess the quality of observational studies based on selection, comparability, and outcome bias. All the studies were assessed on a 1–9 scale, where a higher value indicated a better quality of the study. The quality assessment of the included studies is presented in Table 1, where most included studies had moderate to high methodological quality with scores ranging between 6 and 9. Most of the variability in scores can be attributed to differences in sample size, study design, and the extent of reporting.

### Study characteristics

The features of the studies included in the analysis are presented in Table 2. The studies were conducted in the United States, the United Kingdom, the Netherlands, Austria, Germany, and Australia as the location of the studies with the publication dates spanning between 1996 and 2021. The types of studies employed in the analysis included cross-sectional questionnaire surveys, retrospective and qualitative interview-based studies, with 20–638 patients involved in the studies. The target populations were mainly women of childbearing age, and the mean age of symptom onset ranged from 18 to 23 years, whereas the mean age of diagnosis ranged from 27 to 33 years. The identified factors include patient, provider, and system related to diagnostic delays, time to diagnosis, and the effects of delays on patients’ quality of life.

**Table 2:** Study characteristics of the included studies.

Study title	Authors	Year	Country/region	study design	Sample size	Population characteristics	Setting	Patient-related factors	Provider-related factors	System-related factors
Delay in the diagnosis of endometriosis: A survey of women from the USA and UK	Hadfield et al. (29)	1996	USA, UK	Cross-sectional survey	218	Mean age at symptom onset: 22.39 years; Mean age at diagnosis: 31.80 years	Self-help groups and hospital records	Normalization of symptoms; prolonged pain tolerance	Diagnostic reliance on laparoscopy; misdiagnosis	Longer delays in the USA due to healthcare system differences
Strengths and weaknesses in the diagnostic process of endometriosis	van der Zanden et al. (27)	2021	Netherlands	Qualitative focus group study	23	Age range: 29–45 years; Mean age: 33.9 years	National focus groups	Normalization of symptoms; family influence	Lack of GP knowledge, misdiagnosis, inadequate acknowledgment of symptoms	Limited referral pathways; inconsistent specialist expertise
Women’s experiences of reaching a diagnosis of endometriosis	Ballard et al. (23)	2006	UK	Interview-based qualitative study	32	Age range: 16–47 years; Median age: 32 years	Pelvic pain clinic in Southeast England	Symptoms normalized; embarrassment in disclosing pain	Pain dismissed by GPs; hormonal suppression prescribed; diagnostic reliance on ultrasounds	Long waiting times between primary and secondary care
Diagnostic Delay of Endometriosis in the Netherlands	Staal et al. (24)	2016	Netherlands	Retrospective study	93	Median age at symptom onset: 20 years; Median age at diagnosis: 31 years	Multidisciplinary endometriosis team	Mild symptom presentation; normalization of menstrual pain	GP delays due to symptom misattribution (IBS or stress); over-reliance on contraceptives	Gatekeeping role of GPs; healthcare barriers
Diagnostic Delay for Superficial and Deep Endometriosis in the UK	Ghai et al. (28)	2020	UK	Retrospective cross-sectional study	101	Median age at symptom onset: 20 years; Median age at diagnosis: 31 years	Tertiary referral center	Adolescent presentations increase delays; normalization of pain	Dismissive GP and gynecologist attitudes; symptom misattribution	Public awareness low; referral delays
Gynecologists’ View on Diagnostic Delay in the Netherlands	van der Zanden et al. (26)	2018	Netherlands	Cross-sectional questionnaire study	67 hospitals	Participants included gynecologists from academic and community hospitals	Nationwide study	Patient symptoms trivialized; vague presentations	Limited GP knowledge; symptom misattribution; inconsistent referral practices	Late referrals by GPs; limited collaboration between specialties
Barriers and Facilitators to Timely Diagnosis in Primary Care	van der Zanden et al. (33)	2020	Netherlands	Qualitative focus group study	43 GPs	GPs with varying levels of experience	Medical offices	Perception of symptoms as somatization; cultural differences	Limited GP knowledge; poor guideline adherence; misdiagnosis	Lack of interdisciplinary collaboration
Diagnostic Delay in Austria and Germany	Hudelist et al. (22)	2012	Austria, Germany	Multicenter cross-sectional study	171	Mean age at symptom onset: 21.2 years; Mean age at diagnosis: 32 years	Tertiary referral centers	Normalization of menstrual pain; maternal attitudes	Misdiagnosis (IBS, stress-related pain); limited transvaginal ultrasound use	Access barriers; delayed specialist availability
Etiquette of Endometriosis: Stigma and Concealment	Seear (21)	2009	Australia	Qualitative interview-based study	20	Mean age at symptom onset: 18 years; Mean age at diagnosis: 27 years	Community-based	Menstrual stigma and concealment	Symptom normalization by doctors; dismissal by providers	Social stigma inhibited symptom disclosure
Factors Associated with Time to Endometriosis Diagnosis in the US	Soliman et al. (25)	2017	USA	Cross-sectional online survey	638	Mean age at symptom onset: 23.2 years; Mean age at diagnosis: 27.5 years	Nationwide survey	Symptom normalization; younger age linked to longer delays	Non-specialists caused delays; obstetrics/gynecology (OB/GYN) associated with shorter diagnosis timelines	Geographic disparities; insurance type had limited effect

To enhance readability, diagnostic-process metrics (consultation counts, imaging modality, step-specific delays) have been relocated to Supplementary Table S1.

### Diagnostic delays and contributing factors

**Figure 2:**
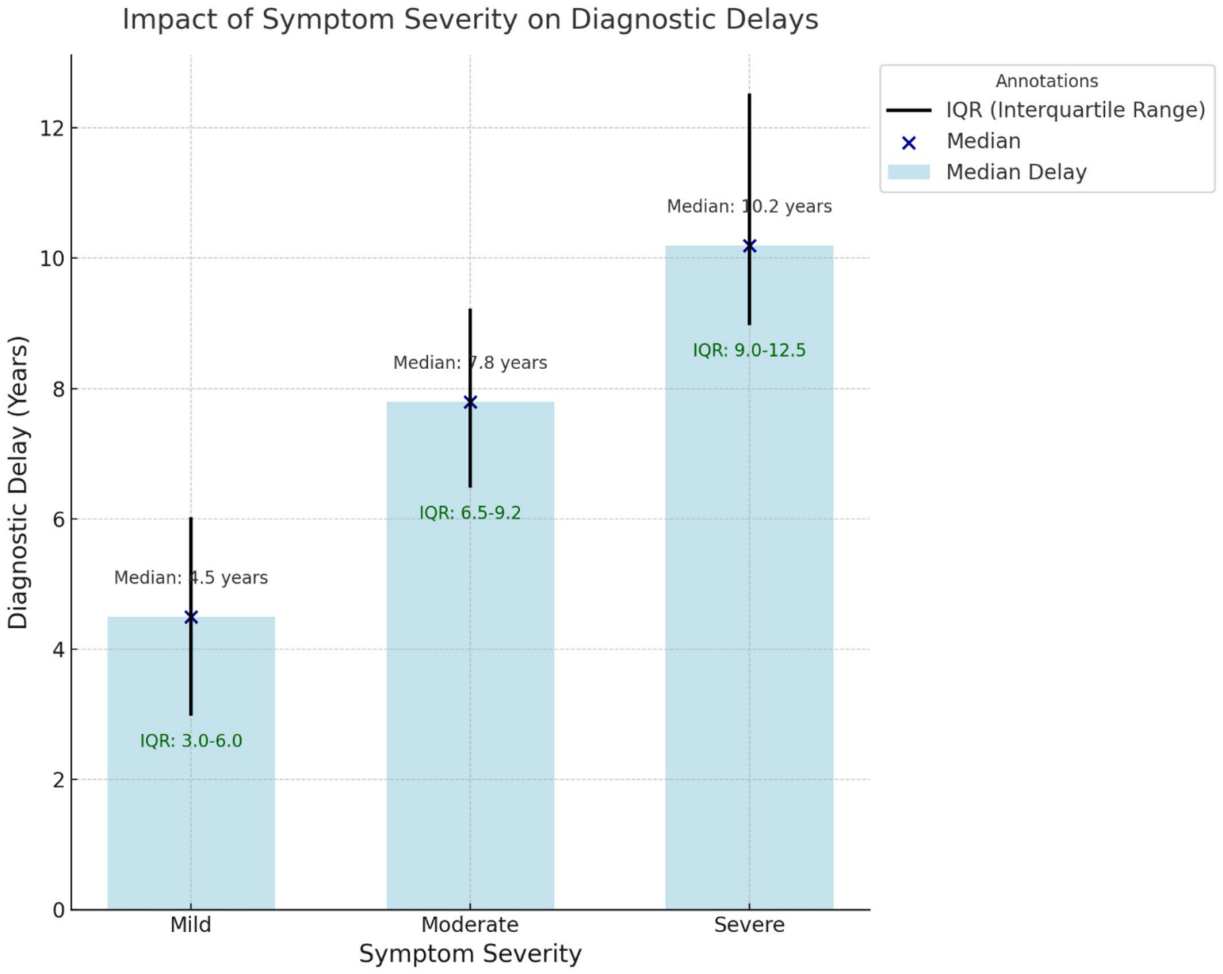
Impact of symptom severity on diagnostic delays. This box-and-whisker plot shows the relationship between symptom severity (mild, moderate, and severe) and median diagnostic delays (in years). The interquartile range (IQR) is represented with whiskers, and individual medians are annotated for clarity. Severe symptoms are associated with the most extended delays.

The evaluation of diagnostic delay revealed that diagnosis of endometriosis was significantly delayed in all the studies included in the review, with the diagnostic time in the most recent studies averaging 4.4 years and the median diagnostic time in the earlier studies exceeding 10 years. These delays were due to patient, provider, and system factors. Patient factors included symptom reporting such as menstrual pain, social culture that attached negative connotations to menstruation, and delay in treatment seeking. For instance, Seear (21) indicated that menstrual stigma hinders early disclosure while Hudelist et al. (22) pointed out that maternal attitudes toward menstruation affect symptom normalization, resulting in delays of more than 14 years. Figure 2 represents the Impact of diagnostic delay on the severity of symptoms.

Provider-related factors were equally important, with many studies describing general practitioners’ (GPs’) misdiagnosis and dismissal of symptoms by non-specialists. Ballard et al. (23) and Staal et al. (24) noted that GPs tend to dismiss symptoms as IBS or stress-related pelvic pain, thereby not referring to specialists. In the same way, Soliman et al. (25) showed that consulting an obstetrician/gynecologist (OB/GYN) reduced diagnostic delay when compared to consulting non-specialists, further emphasizing the value of specialist involvement.

**Figure 3:**
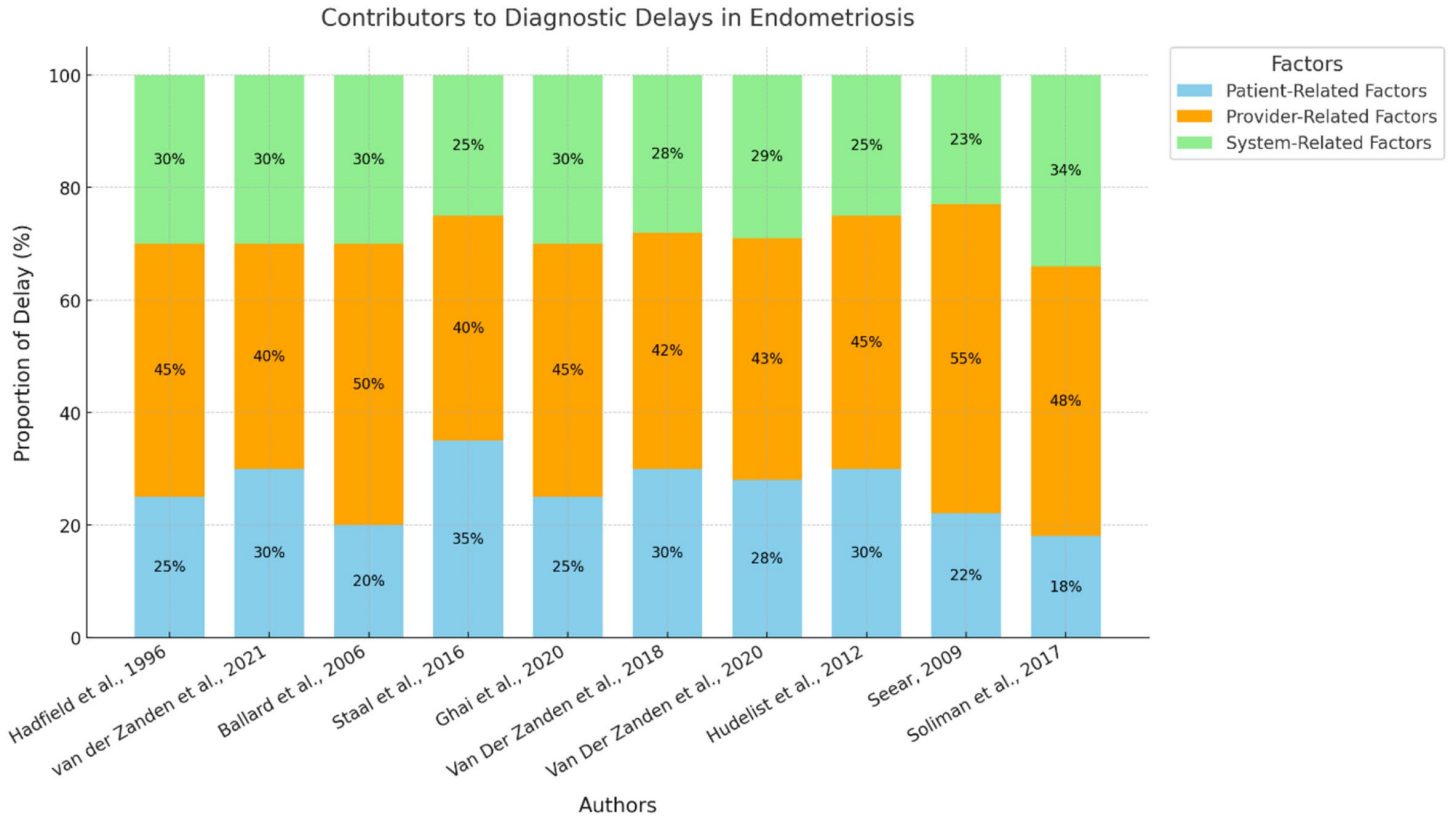
Contributors to diagnostic delays in endometriosis. This stacked bar chart represents the relative contributions of patient-related, provider-related, and system-related factors to diagnostic delays across the 10 included studies. Each study is identified by its author citation, and the chart visualizes the proportional impact of each factor.

Other systemic factors were also found to have influenced diagnostic delays. Research also from the Netherlands (24, 26) pointed out that GPs acted as gatekeepers within the healthcare systems, which led to the delayed access to specialists. Moreover, Ghai et al. (28) pointed out that the public awareness and the referral system in the United Kingdom are not satisfactory, which leads to the delayed diagnosis of up to 11 years for DIE. Figure 3 represents the factors contributing to the delayed diagnosis.

### Impacts of diagnostic delays

**Figure 4:**
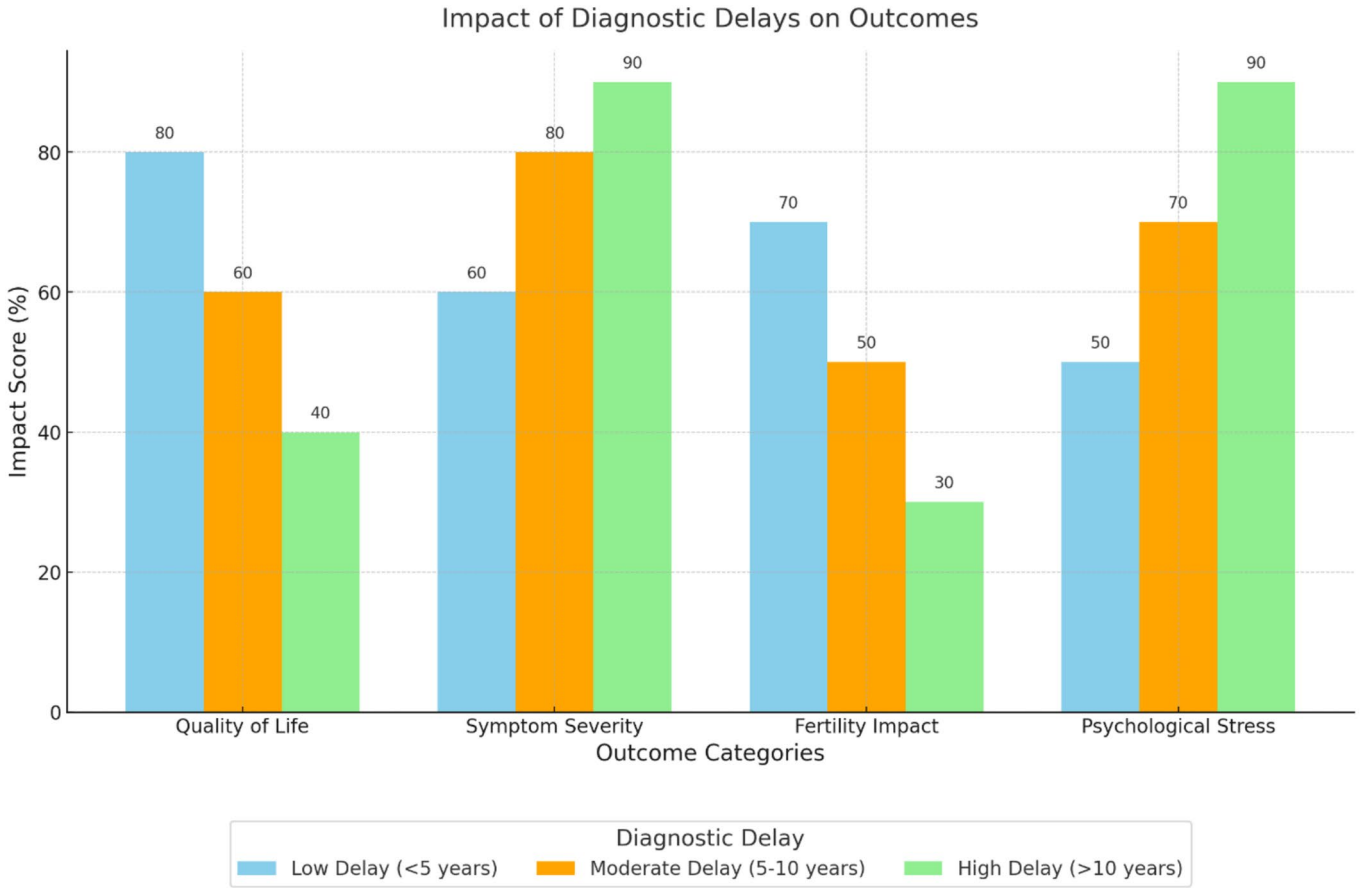
Impact of diagnostic delays on outcomes. This grouped bar chart compares the effects of low, moderate, and high diagnostic delays on various outcomes, including quality of life, symptom severity, fertility impact, and psychological stress. Longer diagnostic delays are associated with more severe impacts across all outcomes, particularly psychological stress and symptom severity.

The delays in diagnosing endometriosis were found to be significantly correlated with negative effects on the physical as well as mental well-being of the patients (Figure 4). Hudelist et al. (22) and Soliman et al. (25) established that delays resulted in worsening of symptoms, higher levels of treatment intervention, and reduced quality of life. Internal outcomes that have been highlighted in the findings are frustration, stress, and impaired self-image. For example, van der Zanden et al. (27) found that participants identified feelings of invalidation and hopelessness because of delayed diagnosis.

Fertility outcomes were another significant aspect that was affected by the delays in diagnostics. According to the study conducted by Ghai et al. (28), female patients with rectovaginal disease who were diagnosed later had more issues with infertility than patients with superficial endometriosis. Such findings highlight the importance of early diagnosis to prevent potential chronic health and reproductive consequences.

### Variations by geography and study design

The duration taken to arrive at the final diagnosis also differed in the studies depending on the geographical region. The time interval between symptom onset and diagnosis was shorter in the US women (25) 4.4 years (mean) as compared to Austria and Germany (22) 10.4 years (median) and the UK (29) 9.41 years (mean). These distinctions may be attributed to disparities in access to healthcare, community awareness, and the availability of diagnostic equipment.

The study designs also affected the reported diagnostic delays. Cross-sectional and qualitative studies provided a broader context of patient and provider experiences; however, the sample size was limited. Retrospective studies reported longer waiting times, which may be attributed to recall bias.

**Figure 5:**
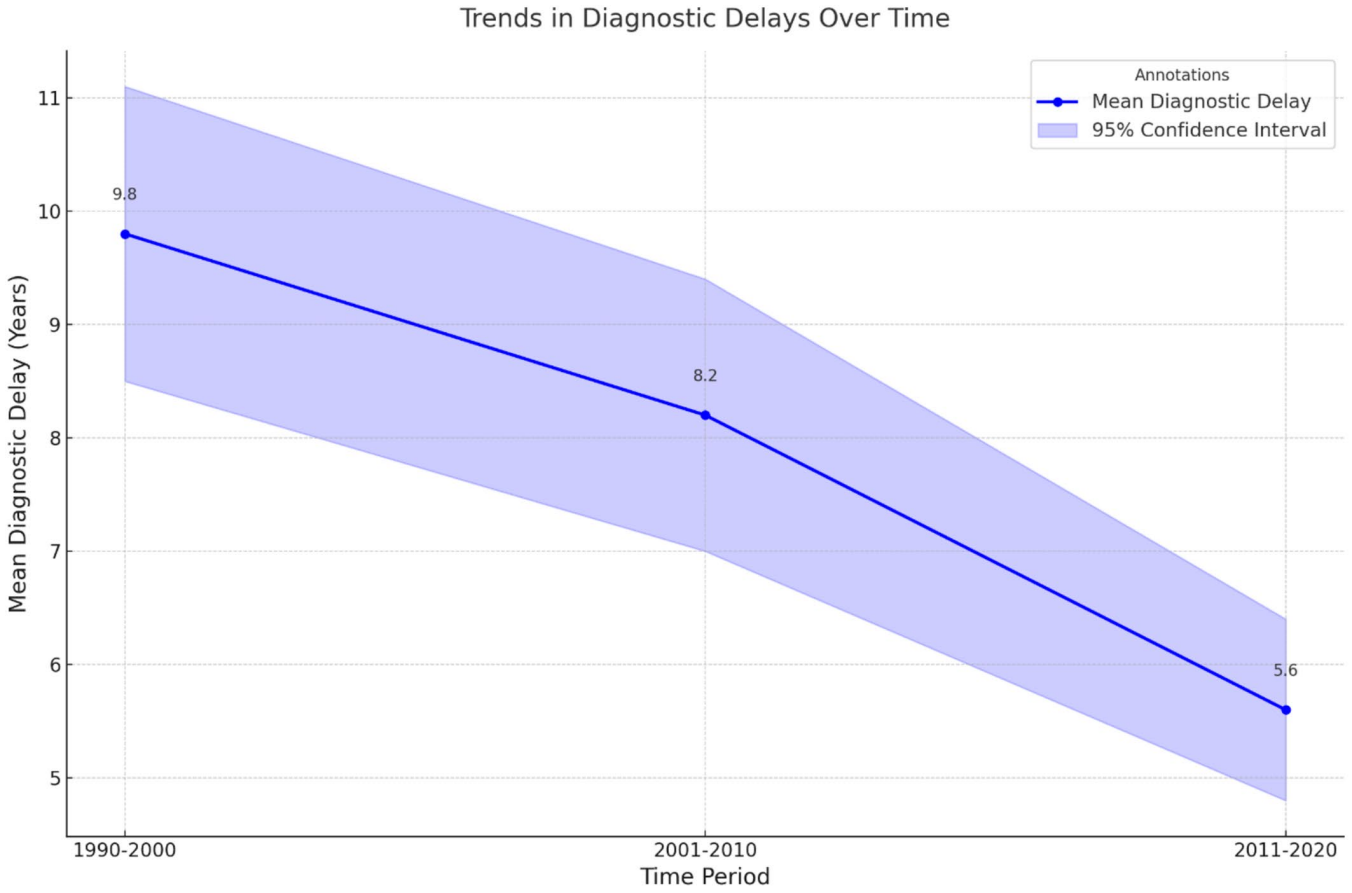
Trends in diagnostic delays over time. This line graph illustrates the mean diagnostic delays (in years) across three time periods (1990–2000, 2001–2010, and 2011–2020). Shaded areas represent the 95% confidence intervals, with annotations indicating the mean delay for each period. A downward trend over time highlights gradual improvements in diagnostic efficiency.

Time was another contributing factor in the delay of diagnosis. Figure 5 illustrates the trend graph illustrating diagnostic delays over time, featuring a clear line graph for mean delays and a shaded area representing the 95% CI.

### Data synthesis

The meta-analysis revealed significant findings regarding the factors contributing to diagnostic delays in endometriosis. Patient-related factors, provider-related factors, and system-related factors were each assessed for their influence on diagnostic timelines, with standardized mean differences (SMD) calculated for each subgroup. Statistical heterogeneity was examined using the I^2^ statistic, and random-effects models were applied to account for between-study variability.

### Patient-related factors

**Figure 6:**
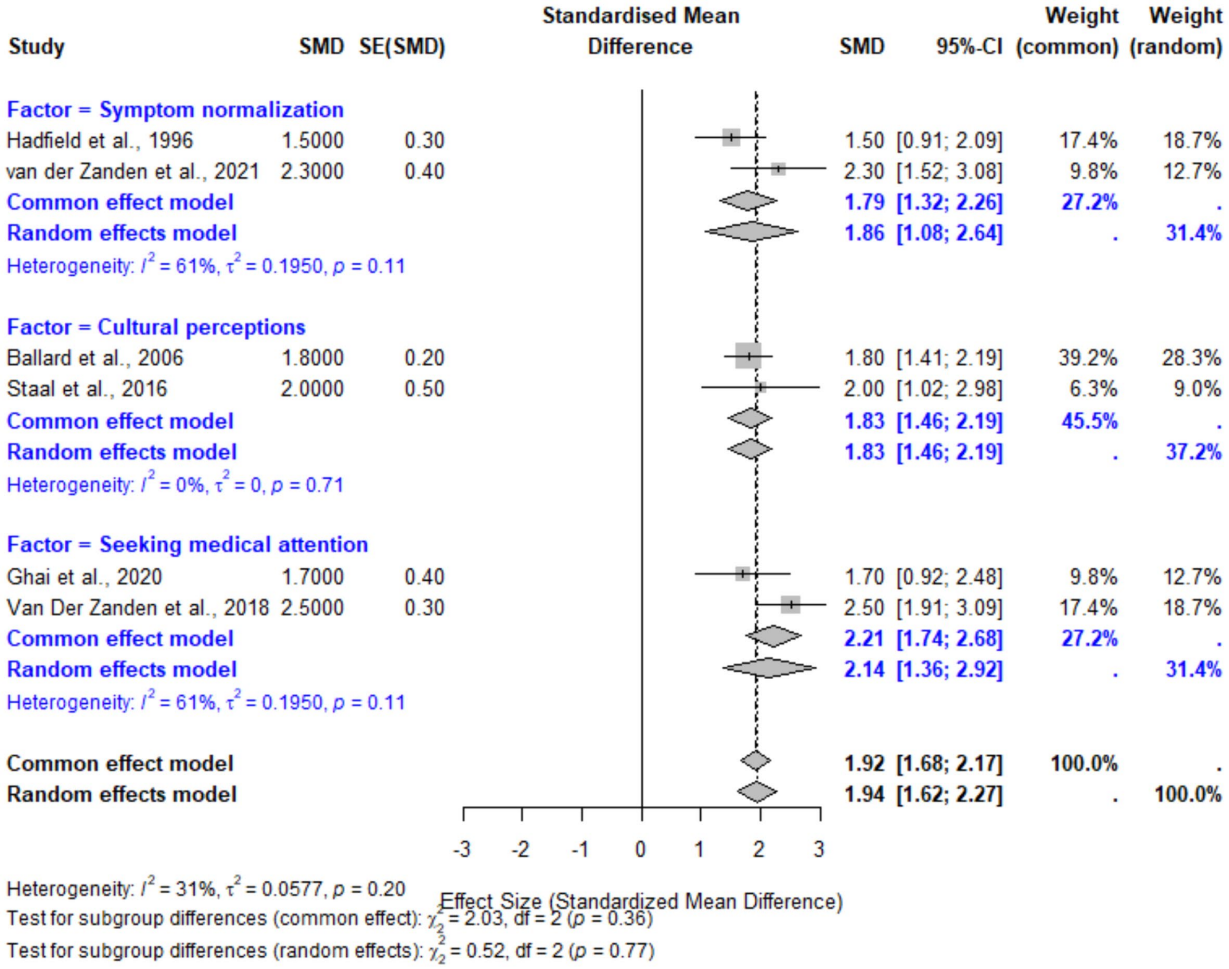
Forest plot for patient-related factors contributing to diagnostic delays in endometriosis. This plot illustrates the pooled effect sizes (SMDs) for subgroups including symptom normalization, cultural and familial perceptions, and delays in seeking medical attention. Random-effects models were employed, with moderate heterogeneity observed (*I*^2^ = 31%).

Patient-related factors, including symptom normalization, cultural and familial perceptions, and delays in seeking medical attention, were found to have a significant overall effect on diagnostic delays. The pooled SMD for these factors was 1.94 (95% CI: 1.62–2.27, *p* < 0.001), indicating a large effect size. Subgroup analyses revealed the highest effect for delays related to seeking medical attention, with an SMD of 2.14 (95% CI: 1.36–2.92), followed by cultural and familial perceptions (SMD: 1.83, 95% CI: 1.46–2.19). Symptom normalization demonstrated a moderate effect size (SMD: 1.86, 95% CI: 1.08–2.64). Heterogeneity was moderate overall (*I*^2^ = 31%), suggesting some variability across studies. These findings highlight the critical need for increased patient education and support to mitigate the normalization and stigma surrounding menstrual symptoms (Figure 6).

### Provider-related factors

**Figure 7:**
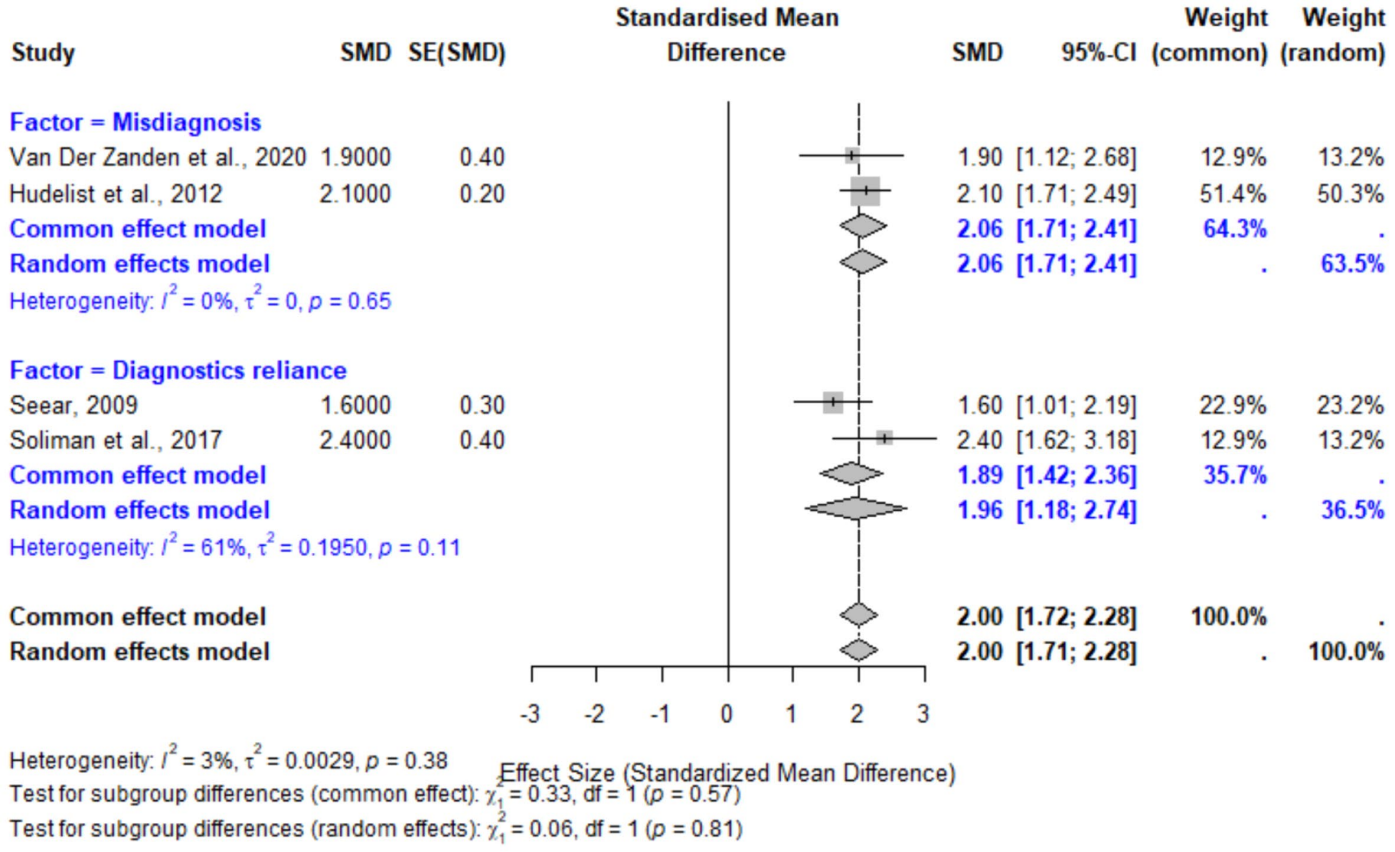
Forest plot for provider-related factors contributing to diagnostic delays in endometriosis. The plot displays pooled effect sizes for misdiagnosis and reliance on non-specific diagnostics. The random-effects model demonstrated low heterogeneity (*I*^2^ = 3%), indicating consistency in the findings.

Provider-related factors, including misdiagnosis or symptom dismissal and reliance on non-specific diagnostics, were also associated with significant diagnostic delays. The pooled SMD for these factors was 2.00 (95% CI: 1.72–2.28, *p* < 0.001). Subgroup analyses revealed slightly higher delays associated with diagnostics reliance (SMD: 1.96, 95% CI: 1.18–2.74) compared to misdiagnosis (SMD: 2.06, 95% CI: 1.71–2.41). Heterogeneity for provider-related factors was low (*I*^2^ = 3%), reflecting consistency across studies. These findings underscore the importance of improved provider education and diagnostic tools to minimize reliance on inaccurate symptom attributions (Figure 7).

### Funnel plot analysis

**Figure 8:**
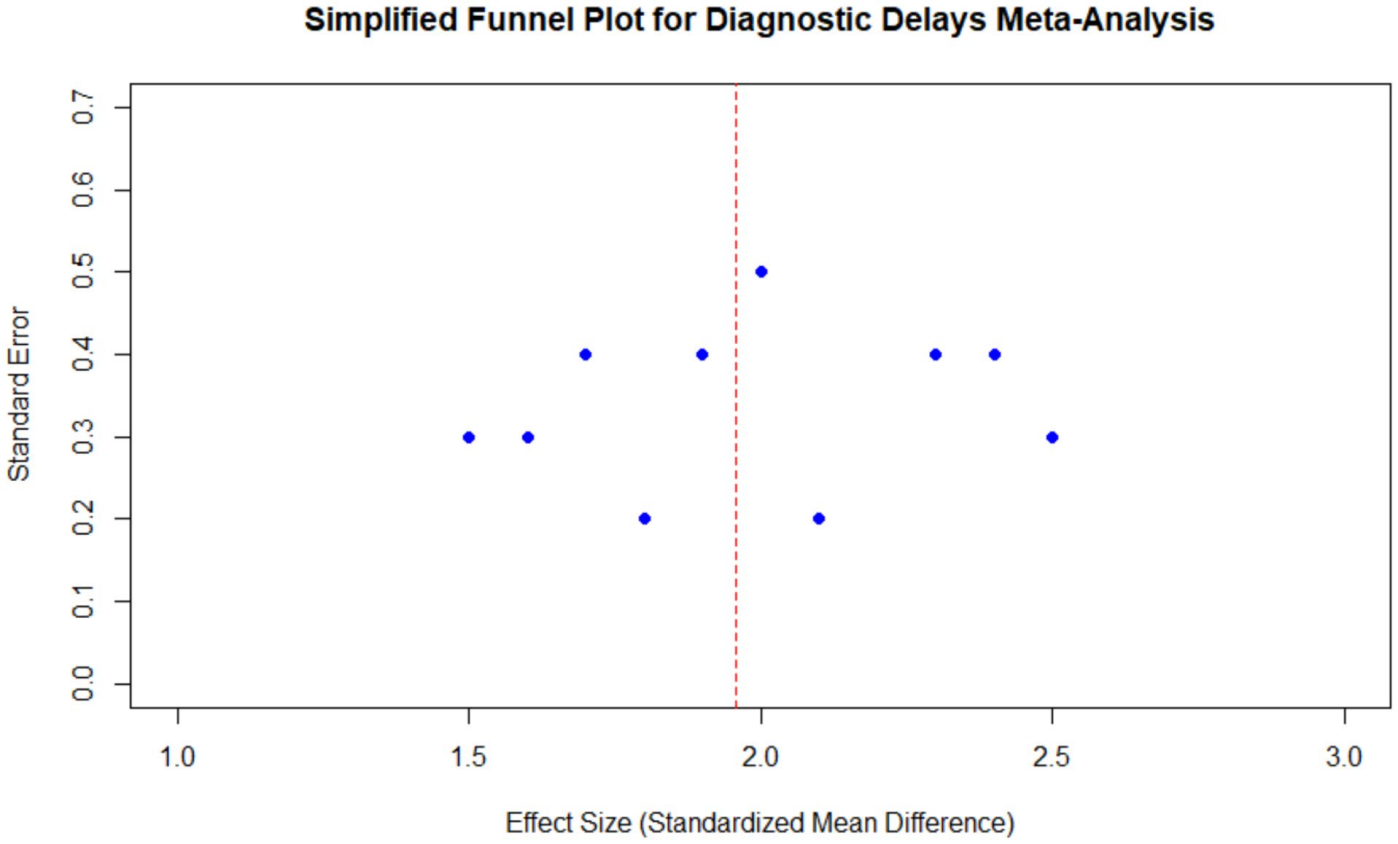
Funnel plot for diagnostic delays in endometriosis meta-analysis. The plot shows the distribution of effect sizes and standard errors for all included studies, centered around the pooled effect size. The red dashed line represents the overall pooled effect, and the symmetric distribution indicates no significant publication bias.

The funnel plot of all the included studies showed that the points were symmetrically distributed around the estimate of the pooled effect size, indicating no indication of publication bias. Egger’s regression test for asymmetry confirmed this result, with a non-significant *p*-value (*p* = 0.57). The red dashed line represents the overall pooled effect, and the distribution of studies suggests that the results are robust and not influenced by small-study effects (Figure 8).

## Discussion

The systematic review and meta-analysis of the endometriosis diagnostic delay demonstrates that the diagnosis and treatment of such illness is a lengthy process, and is associated with numerous factors, which impact the lives of millions of women worldwide. However, even in the current century, with increased awareness of the disease and medical advancements, significant delays are observed, and diagnosis times vary between 4.4 years and more than 10 years in the studies under review. The conclusions drawn have highlighted the need for structural interventions in healthcare organizations, patient awareness, and diagnostic procedures to address these delays.

### Delays in diagnosis: a persistent problem

The diagnostic delays described in this review are not surprising, as the literature has long documented the difficulties in early endometriosis diagnosis for decades. Thus, cultural acceptance of menstrual pain by the patients and the providers was another dominant pattern found in the studies such as Seear (21) and Hudelist et al. (22). Lack of patient education and cultural taboo regarding the female reproductive system also contributed to the delay in seeking medical attention among such patients. This study highlights the importance of social cognition and education in reducing the time to diagnosis.

Another vital cause was provider-related factors. Ballard et al. (23) and Staal et al. (24) showed that general practitioners and other non-specialists frequently either denied or explained away symptoms. Symptoms of IBS, chronic appendicitis, or some forms of psychological disorders were often misinterpreted, leading to either a too-long diagnostic process or unwarranted treatments and further suffering. In the study by Soliman et al. (25), the involvement of OB/GYN consultants was highlighted as it added value to the case and resulted in shorter diagnosis times. The results of this study suggest that increasing the awareness of primary care providers concerning the symptoms of endometriosis and their capacity to recognize them can considerably reduce the time to diagnosis.

### Limitations and strengths

A further limitation is that four of the 10 studies predate 2010. Nevertheless, sensitivity analyses excluding these studies yielded effect sizes within the original 95% CI, suggesting that the core barriers—symptom normalization, misdiagnosis, and gate-keeping—remain operative in today’s clinical environment. Another limitation is the relatively small number of included studies despite a comprehensive search strategy. The stringent inclusion criteria, focusing on quantitative assessments of diagnostic delays, limited the pool of eligible studies but ensured methodological rigor in the analysis.

The strengths of this systematic review include its comprehensive approach to categorizing contributing factors into patient, provider, and system-related domains, which provides a nuanced understanding of where interventions might be most effective. The use of standardized mean differences allowed for meaningful comparisons across diverse study designs and populations.

### Comparisons with recent work

Our findings on diagnostic delays should be considered in the context of recent advances in treatment approaches for endometriosis. The feasibility of advanced minimally invasive surgical techniques for treating deep endometriosis using new robotic systems has been demonstrated, highlighting the importance of timely diagnosis in leading to effective therapeutic interventions (30). Their case series reported significant symptom improvement following surgery, with mean dysmenorrhea scores decreasing from 9.50 to 1.7 (*p* = 0.001) and chronic pelvic pain from 8.8 to 3.20 (*p* = 0.001) after surgical intervention.

In a comprehensive meta-analysis comparing robotic-assisted versus conventional laparoscopic surgery for deep endometriosis across 14 studies including 2,709 patients, no significant differences in complication rates between the approaches were found. However, the potential benefits of integrating robotic platforms with new diagnostic technologies were emphasized (31, 32). These innovations in treatment underscore the importance of minimizing diagnostic delays, as patients can only benefit from these advanced therapeutic options after receiving a definitive diagnosis. Additionally, the ongoing evolution of surgical techniques, such as the en-block butterfly excision for posterior compartment deep endometriosis, as described in a video article, further emphasizes the critical need for timely diagnosis (30). These advanced surgical approaches become viable options only after patients have navigated the often-lengthy diagnostic journey described in our review. The integration of improved diagnostic pathways with these evolving treatment modalities represents a promising direction for comprehensive endometriosis care (34, 35).

### Systemic barriers and geographic variations

Healthcare structures, including access to services, networking, and interdisciplinary care coordination, were other organizational factors that contributed to diagnostic inertia. Two research studies conducted in the Netherlands (24, 26) highlighted that such a gatekeeping role of GPs played a part in the construction of barriers to specialist care. Similarly, Ghai et al. (28) and Hadfield et al. (29) found that the time taken was longer in countries with a more fragmented or less accessible healthcare system, like the USA and UK. They suggest that the process of information exchange between the referrer and the patient should be made more efficient and the lines of care coordination should be tightened.

Other factors that could lead to regional differences in the time it takes to diagnose include variations in health facilities and public information. While the United States reported a smaller waiting time of less than 1 year (25), probably due to easy access to OB/GYNs and better diagnostic facilities, other European countries like Austria and Germany (22) had a median delay of more than 10 years. This disparity suggests that the distribution of healthcare resources than their availability, could be the key driver of diagnosing productivity.

Notably, our review identified a significant gap in data from low-and middle-income countries (LMICs), where resource constraints and cultural factors may further exacerbate diagnostic delays. This underscores the need for context-specific interventions that address both healthcare system capacity and cultural factors that may differ substantially from those in high-income settings.

### Implications for patient outcomes

The delays in diagnosis have drastic effects on disease progression, the quality of life for patients, and their reproductive capabilities. van Laarhoven et al. (36) review and Hudelist et al.’s (22) and Ghai et al.’s (28) meta-analyses indicated that diagnostic delays were associated with higher symptom severity and lower quality of life. These outcomes are quite worrisome, especially given that early intervention can ease symptoms and enhance future health. Other psychological effects such as frustration, hopelessness, and poor self-image were also reported by van der Zanden et al. (27) and Ballard et al. (23) on delayed diagnosis.

Another important facet that is affected by diagnostic delays is fertility outcomes. Ghai et al. (28) revealed that women with rectovaginal endometriosis had significantly higher rates of fertility impairment compared to those with superficial endometriosis. Consequently, it is critical to identify the connection between delayed diagnosis and reproductive outcomes so that fertility can be protected and complications avoided in the future.

### Role of diagnostic methods

The review also discusses the changes in diagnostic methods and their impact on diagnostic time. The conventional approach, based on laparoscopic surgery, which is the best practice, took longer time as highlighted in Hudelist et al. (22). However, Soliman et al. (25) note that the utilization of transvaginal ultrasound and Magnetic resonance imaging (MRI) in diagnosing epithelial ovarian cancer (EOC) has reduced the time it takes to diagnose the disease in the recent past. This shift requires the use of modern imaging modalities and evidence-based interventions in practice to expedite diagnosis while maintaining efficiency.

### Recommendations for reducing delays

To address the issue of the delay in endometriosis diagnosis and its chronic nature, there is a need to develop an intervention that will involve everyone in the system. First, it is possible to launch information and awareness campaigns targeting adolescents, young women, and their families with the purpose of demystifying the concept of menstruation and encouraging them to seek help earlier. Other works, including those by Seear (21) and Soliman et al. (25) suggest that prejudice should be eradicated, and people should be made aware of the symptoms of endometriosis.

Second, there is a need to enhance the provider education. The educational interventions aimed at GPs and non-specialists should focus on raising awareness of the symptoms of endometriosis, the possibility of differentiating it from other pathologies, and the importance of referral. The referral criteria and diagnostic pathways should also be part of the guidelines to primary care providers as suggested by Staal et al. (24) and van der Zanden et al. (26).

Third, there is need for system improvements in the patient referral process and improving the existing relations between the departments. The increase of OB/GYN specialists in primary care networks and the creation of full-service care teams may help enhance diagnostic accuracy and reduce the time to detection. However, it is also possible that optimizing the application of general diagnostic protocols and the availability of non-invasive diagnostic aids could improve the diagnostic phase.

### Future directions

Next-generation diagnostic pathways will likely harness (i) machine-learning classifiers that integrate electronic-health-record metadata with laparoscopic imaging, (ii) circulating microRNA or cell-free DNA (cfDNA) panels as non-invasive biomarkers, and (iii) polygenic-risk–based screening algorithms. Early work combining deep convolutional networks with multispectral laparoscopy has achieved area under the curves (AUCs) > 0.90 in differentiating deep-infiltrating endometriosis. Large multiomic consortia and federated learning pipelines could further shorten the diagnostic interval while preserving patient privacy.

Further studies are also needed regarding the antecedents of diagnostic delay and the effectiveness of strategies aimed at addressing it, such as health promotion campaigns and clinical continuing medical education programs. Finally, the development of algorithms that can aid in identifying high-risk patients and thereby prioritize diagnostic assessments may improve the results.

## Conclusion

This systematic review and meta-analysis reveal various factors that contribute to diagnostic delays for endometriosis and the negative outcomes that follow. Reducing such delays requires multistakeholder efforts that include patient education, clinician training, and policy adjustments. To this end, the application of these strategies will assist the healthcare systems in diagnosing women with endometriosis at an early stage and alleviating the suffering of these women by reducing the impact of this severe disease on their lives.

## Data Availability

The original contributions presented in the study are included in the article/Supplementary material, further inquiries can be directed to the corresponding author.

## Footnotes

^1^ The R Foundation for Statistical Computing Development Team: R Development Core Team Website: https://www.r-project.org/ Version 4.2.3 Release Date: March 15, 2023.
