# Supplementary material for: Factors contributing to the delayed diagnosis of endometriosis—a systematic review and meta-analysis
Source: Front Med (Lausanne). 2025 Jul 22;12:1576490. doi: 10.3389/fmed.2025.1576490 (PMC12321876; doi:10.3389/fmed.2025.1576490)
Supplement: Supplementary file 1 [file Table_1.docx]

# Supplementary Table A1: Diagnostic process metrics for included studies

| **Study (Author, Year)** | **Diagnostic Timeframe** | **Number of Consultations Before Diagnosis** | **Diagnostic Tests Used** | **Average Time Between Steps** | **Reported Outcomes** | **Statistical Measures** | **Other Key Findings** |
| --- | --- | --- | --- | --- | --- | --- | --- |
| Hadfield et al., 1996 | UK: 7.96 years; USA: 11.73 years | Not specified | Laparoscopy | Not specified | Delays cause prolonged suffering and no link between symptom severity and diagnosis | Mean delay: 9.41 years; significant UK-USA difference (p < 0.01) | Younger age groups experienced longer delays |
| van der Zanden et al., 2021 | Mean delay: 8.5 years; GP to referral: 61 months; Symptoms to GP: 33 months | Not reported | Laparoscopy or MRI | GP to gynecologist referral: 61 months | Delays caused frustration; negative body image; psychological stress | Mean delay: 8.5 years; delays longer for referral step | Emphasis on professional acknowledgment reducing delays |
| Ballard et al., 2006 | Median delay: 8 years; IQR: 6–12 years; Range: 2 months–22 years | Multiple GP visits required | Laparoscopy | 6–48 months between primary and secondary care | Diagnosis provided validation of symptoms; improved societal acceptance | Diagnostic delays median: 8 years; IQR: 6–12 years | Greater awareness needed for both patients and healthcare providers |
| Staal et al., 2016 | Median total delay: 7.4 years (89 months); GP delay: 35 months; Specialist to diagnosis: 5 months | Not reported | Surgery or MRI | Symptom onset to GP consultation: 2.3 years; GP to gynecologist referral: 2.7 years | Diagnostic delays linked to missed treatments and psychological distress | Subfertility-related symptoms shorter (21 months) vs pain-related symptoms (100 months) | Enhanced GP recognition needed for reducing referral delays |
| Ghai et al., 2020 | Median total delay: 8 years; Rectovaginal disease delay: 11 years; Superficial: 5 years | Median GP visits: 5 | Laparoscopy | Symptom onset to GP: 1 year (median); GP to diagnosis: 7 years | Severe impact on quality of life; delays increased for rectovaginal disease | Earlier symptom onset linked to longer delays (Spearman's Rank: –0.63, p<.01) | Public and professional education essential |
| Van Der Zanden et al., 2018 | Median delay: 42 months (3.5 years) | Not reported | Laparoscopy; Ultrasound; MRI | Not reported | Delays negatively affected quality of life; recommended improved professional collaboration | High adherence to ESHRE guidelines | Educational campaigns for patients and providers recommended |
| Van Der Zanden et al., 2020 | Not quantified | Multiple consultations often required | Rare primary care diagnostics; referrals after significant delays | Not reported | Delays linked to missed treatment opportunities and psychological impacts | No specific statistical measures | Interdisciplinary collaboration and greater awareness crucial |
| Hudelist et al., 2012 | Median delay: 10.4 years; GP consultation to diagnosis: 7.7 years | Mean gynecologists consulted: 3.4 | Laparoscopy; limited transvaginal ultrasound | Symptom onset to GP: 2.3 years; GP to specialist: 2.7 years | Prolonged delays led to missed early treatments and psychological distress | Misdiagnosis linked to longer delays (11.5 vs. 7.4 years, p<.01) | Maternal attitudes toward menstruation increased delays (14.6 vs. 9.7 years, p<.01) |
| Seear, 2009 | Mean delay: 9 years | Not reported | Laparoscopy | Not reported | Delays caused worsened symptoms and psychological stress | Not applicable for qualitative study | Public awareness campaigns targeting social stigma recommended |
| Soliman et al., 2017 | Mean delay: 4.4 years; Symptom to consultation: 2.4 years; Consultation to diagnosis: 2.0 years | Not reported | 49% surgical diagnosis; 51% nonsurgical | Symptom onset to consultation: 2.4 years; Consultation to diagnosis: 2.0 years | Delays linked to symptom severity; Younger women experienced greater delays | OB/GYN diagnosis quicker (21.5 months) vs non-specialists (40.3 months, p=0.041) | Education programs for adolescents and non-specialist physicians recommended |
